# Patients With Systemic Reaction to Their Hernia Mesh: An Introduction to Mesh Implant Illness

**DOI:** 10.3389/jaws.2023.10983

**Published:** 2023-01-30

**Authors:** Negin Fadaee, Desmond Huynh, Zayan Khanmohammed, Laura Mazer, Isabel Capati, Shirin Towfigh

**Affiliations:** ^1^ California Health Sciences University College of Osteopathic Medicine, Clovis, CA, United States; ^2^ Cedars-Sinai Medical Center, Los Angeles, CA, United States; ^3^ Department of Surgery, University of California, Berkeley, Berkeley, CA, United States; ^4^ Higher Ground Education, Lake Forest, CA, United States; ^5^ Beverly Hills Hernia Center, Beverly Hills, CA, United States

**Keywords:** mesh, allergy, mesh reaction, mesh removal, mesh-ASIA, hernia mesh, mesh implant illness, Shoenfeld’s syndrome

## Abstract

In our practice, we have noticed an increased number of patients requiring mesh removal due to a systemic reaction to their implant. We present our experience in diagnosing and treating a subpopulation of patients who require mesh removal due to a possible mesh implant illness (MII). All patients who underwent mesh removal for indication of mesh reaction were captured from a hernia database. Data extraction focused on the patients’ predisposing medical conditions, presenting symptoms suggestive of mesh implant illness, types of implants to which reaction occurred, and postoperative outcome after mesh removal. Over almost 7 years, 165 patients had mesh removed. Indication for mesh removal was probable MII in 28 (17%). Most were in females (60%), average age was 46 years, with average pre-operative pain score 5.4/10. All patients underwent complete mesh removal. Sixteen (57%) required tissue repair of their hernia; 4 (14%) had hybrid mesh implanted. Nineteen (68%) had improvement and/or resolution of their MII symptoms within the first month after removal. We present insight into a unique but rising incidence of patients who suffer from systemic reaction following mesh implantation. Predisposing factors include female sex, history of autoimmune disorder, and multiple medical and environmental allergies and sensitivities. Presenting symptoms included spontaneous rashes, erythema and edema over the area of implant, arthralgia, headaches, and chronic fatigue. Long-term follow up after mesh removal confirmed resolution of symptoms after mesh removal. We hope this provides greater attention to patients who present with vague, non-specific but debilitating symptoms after mesh implantation.

## Introduction

Mesh implantation for hernia repair has become standard practice for the majority of hernia repairs (1). Mesh-based hernia repairs have been shown to be a durable solution, however, postoperative complications, such as chronic postoperative pain, remain a concern. Chronic pain following mesh inguinal hernia repair is either neuropathic and/or nociceptive (2). In our practice, which specializes in the management of complications after herniorrhaphy, we have noticed an increasing incidence of a new cause of complications after mesh-based hernia repairs: a systemic reaction to the mesh material (3).

To date, there have been few investigations into the inflammatory response to mesh (4, 5). These show variability in patients’ responses to mesh and suggest there is a group of patients who are “high responders.” This subpopulation exhibits a significantly more virulent immunologic response to mesh in comparison to their peers (6). This inflammatory response to implant material has been termed “autoimmune/inflammatory syndrome induced by adjuvants (ASIA)” or “Shoenfeld’s syndrome” after Dr. Yehuda Shoenfeld who first acknowledged this reaction (7).

ASIA/Shoenfeld’s syndrome may occur as a reaction to any implant. Given that this syndrome is considered to occur only in a small subset of patients, there is limited *in vivo* data and even less description of the clinical consequences of these reactions. Only one study has described ASIA in a population of patients after mesh implantation, such as for hernia repair and pelvic organ prolapse surgery (8). Others have shown ASIA in patients after silicone breast implantation (9–14).

We have an interest to evaluate ASIA specifically among patients undergoing hernia repair surgery. We chose the term mesh implant illness (MII) to refer to the subset of patients with ASIA whose illness stems from a systemic reaction to their mesh implant. This terminology stems from the well established term, breast implant illness (BII), which refers to the subset of patients with reactions to breast implants. We reviewed MII patients’ clinical findings and followed their outcomes after mesh removal, with the goal of developing a comprehensive plan of care for patients with MII.

## Materials and Methods

Records were reviewed from all patients who underwent implant removal following a hernia repair at a single surgeon center (ST) between August 2013 and June 2020. Data was extracted from a prospectively maintained hernia database.

**FIGURE 1:**
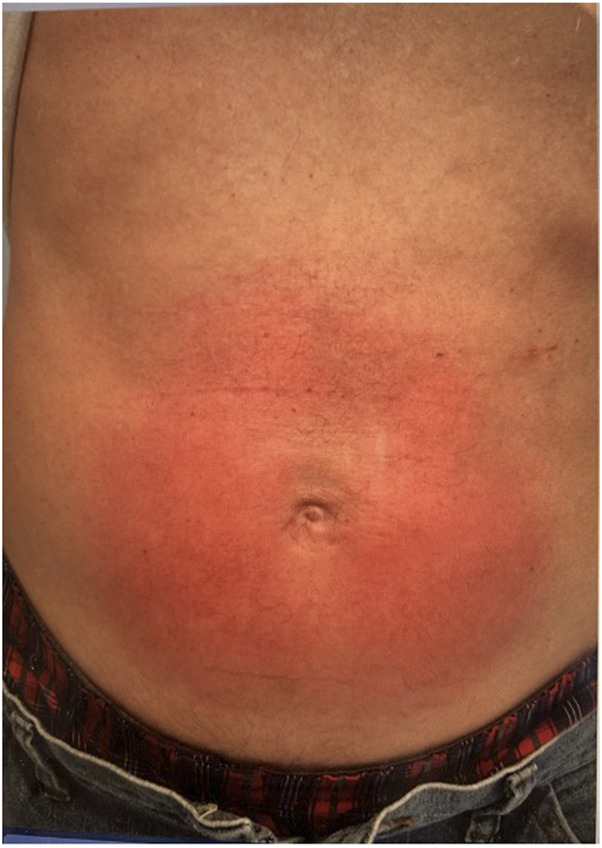
Abdominal wall macular rash after open ventral hernia repair with 4.3 cm round onlay mesh. This is a direct dermatologic reaction to the mesh and not considered a systemic MII.

A systemic mesh reaction captured as MII was defined as any post-herniorrhaphy illness that was not locally neuropathic or nociceptive. All attempts were made to rule out other causes of their illness, which typically included gastroenterologic, urologic, gynecologic, orthopedic, rheumatologic, allergic, immunogenic, dermatologic, neurologic, and/or infectious workups (Figure 1). Patients with suspected chronic mesh infection, who had findings of inflammation on preoperative imagine or abnormalities in blood testing suggestive of chronic infection (for example, abnormal CBC, differentials, ESR, other inflammatory markers) were not included in this population. Data collection included patient demographics, medical history, surgical history, allergy history, family history, presenting symptoms, hernia type, operative details, implant material removed, and postoperative outcomes. Patients were followed up in person and by phone. Short-term follow-up is defined as within 30 days after surgery.

Statistical analyses included Fisher’s exact and Chi-square test.

## Results

Over a span of almost 7 years, 191 of 847 (23%) hernia-related operations involved implant removal. Of these, 165 (86%) patients had one or more meshes removed. Others involved suture and/or tack removal only and were excluded from our analysis. We divided our mesh removal population into two groups: Patients with MII and those without MII. Among patients who underwent mesh removal, 28 (17%) had mesh removed for the postoperative diagnosis of probable MII, while 137 (83%) had mesh removed for other reasons such as pain, meshoma, infection, neuralgia, and/or hernia recurrence (Table 1). Among the 28 patients with a likely MII, 16 (57%) were female, average age was 46 years (range 22–68), and average BMI was 24.8 kg/m^2^ (range 17.64–32.80) (Table 2). Seven MII patients (25%) had their original hernia repair and mesh placement performed by us.

**TABLE 1:** Operative details for patients that underwent mesh removal due to mesh implant illness (MII) or other reasons (non-MII).

	MII	Non-MII	*p*
	N = 28	N = 137	
Indication for removal, N (%)			
Pain	23 (82%)	101 (74%)	NS
Recurrence	8 (29%)	46 (34%)	NS
Neurectomy	6 (21%)	34 (25%)	NS
Neuralgia	5 (18%)	11 (8%)	NS
Meshoma	3 (11%)	54 (39%)	**0.003**
Numbness	2 (7%)	2 (1%)	NS
Infection	1 (4%)	25 (18%)	NS
Index Surgical Approach^a^			
Open	13 (47%)	82 (60%)	NS
Laparoscopic	13 (43%)	43 (31%)	NS
Robotic	3 (13%)	7 (5%)	NS
Time to Mesh Removal			
Average (range)	3.5 years (3 months - 26 years)	4 years (12 days - 27 years)	NS
Mesh Removal Approach^b^			
Robotic	14 (50%)	43 (31%)	NS
Open	10 (36%)	71 (52%)	NS
Laparoscopic	4 (14%)	21 (15%)	NS
Combination	0 (0%)	2 (1%)	NS

^a^ Some patients had multiple prior repairs.

^b^ Some patients had multiple meshes removed.

**TABLE 2:** Demographics of patients that underwent mesh removal due to mesh implant illness (MII) or other reasons (non-MII).

	MII	Non-MII	*p*
	N = 28	N = 137	
Age, mean (range)	46 (22–69)	54 (21–81)	**0.005**
Sex, male (%)	12 (43%)	84 (61%)	NS
BMI, kg/m^2^, mean (range)	24.8 (17.6–32.8)	26.8 (17.8–43.9)	NS
^a^History of Autoimmune, Yes (%)	3 (11%)	8 (6%)	NS

^a^ Some patients have multiple autoimmune disorders.

**FIGURE 2:**
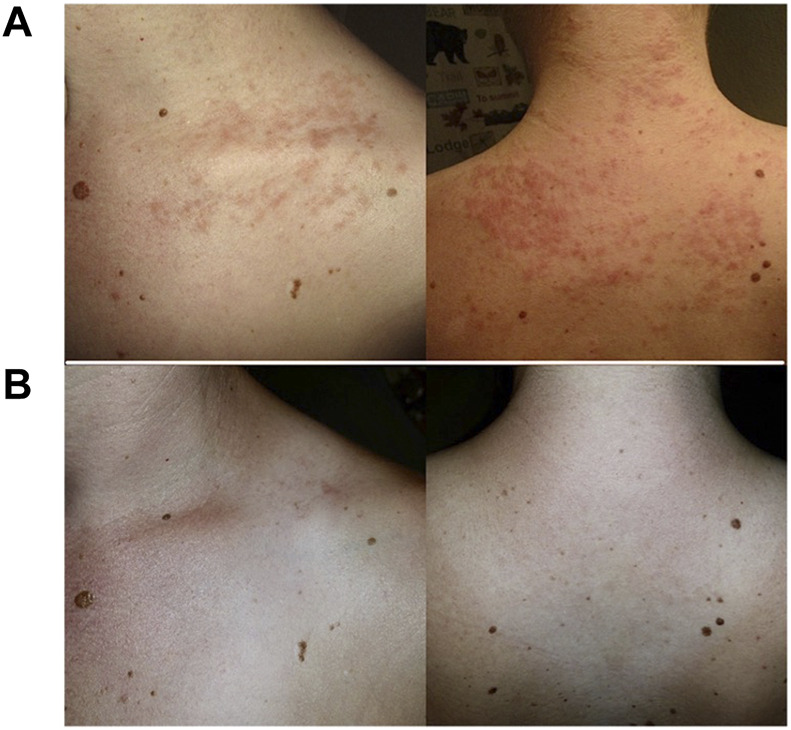
Neck and back maculopapular rashes **(A)** after inguinal hernia repair with onlay mesh and **(B)** resolution after mesh removal.

All of the patients with suspected MII had at least one of the following new symptoms as part of their syndrome: chronic fatigue (23, 82%), bloating with or without nausea (18, 64%), local swelling (16, 57%), joint pain (14, 50%), rash or erythema (13, 46%), headaches (12, 43%), fevers (9, 32%), and fibromyalgia (3, 11%) (Table 3). Of those with new and inexplicable rashes, 8 (62%) had a body rash distant from the area of mesh implant, e.g. along the neck, chest and back (Figure 2A). Symptoms began shortly after the mesh implant. Seven patients (25%) reported immediate start of symptoms, i.e., within days of their hernia surgery with mesh. Two patients (7%) reported symptoms within weeks, and 4 (14%) reported symptoms within 4 months postoperatively. The majority (23, 82%) of patients also complained of pain at the surgical site. The average pre-operative pain score was 5.4/10 (range 1–10).

**TABLE 3:** Symptoms prior to mesh removal in patients with suspected mesh implant illness (MII).

Symptoms, N (%)	MII
	N = 28
Fatigue	23 (82%)
Bloating	18 (64%)
Swelling	16 (57%)
Joint Pain	14 (50%)
Rash	13 (46%)
Full Body	8 (62%)
Localized	5 (38%)
Headache	12 (43%)
Fevers	9 (32%)
Fibromyalgia	3 (11%)

Three patients with suspected MII (11%) had a known personal history of an autoimmune and/or inflammatory disorder prior to the mesh implantation. An additional 3 patients (11%) had a family history of autoimmune and/or inflammatory disorder without themselves having known autoimmune and/or inflammatory disorder. Postoperatively, after initial mesh implantation, 12 more patients (43%) were diagnosed with autoimmune and/or inflammatory disorders, for a total of 15 (54%) with a personal history. These included: Hashimoto’s thyroiditis (3), Chronic Fatigue Syndrome (5), Fibromyalgia (2), Lyme Disease (2), Ehlers-Danlos Syndrome (1), Autoimmune Urticaria (1), Mast Cell Activation Syndrome (1), Lupus Erythematosus (1), Common Variable Immunodeficiency (1), and Lichen Planus (1). Eleven (39%) had multiple allergies and sensitivities to medications, foods, implants and environmental pathogens. In the non-MII group, 8/137 (6%) patients had a known personal history or autoimmune and/or inflammatory diagnosis prior to their mesh removal. These included Sjögren’s Syndrome (3), fibromyalgia (2), Lupus Erythematosus (1), Grave’s Disease (1), Celiac Disease (1), Common Variable Immunodeficiency (1), Fibromyalgia (1), Ulcerative Colitis (1) and Crohn’s Disease (1).

All patients with suspected MII underwent extensive testing to help explain their new postoperative symptoms, including evaluations by gastroenterologists, neurologists, dermatologists, allergy/immunologists, orthopedic surgeons, urologists, and/or rheumatologists. This included blood testing to rule out disorders other than MII. All patients with MII had normal blood testing as it related to inflammatory and autoimmune markers. Seven patients underwent preoperative allergy and immunology evaluation, which included skin patch testing against various sutures and meshes.

All 28 patients with suspected MII had one or more mesh implants removed. The most common type of mesh material removed was polypropylene (20, 71%) (Table 5). All patients underwent complete mesh removal. This occurred on average 3.5 years after mesh implantation (range 3 months–26 years). Patients had mesh removed from the pelvis (20, 71%) and from the anterior abdominal wall (8, 29%) *via* robotic (14, 50%), open (10, 36%) or laparoscopic (5, 14%) approach. In general, meshes placed as an onlay were removed *via* open technique and those placed as a sublay were removed *via* laparoscopic or robotic approach. Our techniques have been previously described (15, 16).

Sixteen (57%) of the mesh removals among patients with MII were performed as an outpatient. Most (21/28, 75%) operations were performed under general anesthesia. Nearly half (12/28, 43%) of the operations were performed as an inpatient with an average length of stay of 2.8 nights (range 2–5). Upon mesh removal, 16 (57%) patients underwent tissue-based hernia repair without mesh, 7 (25%) patients had complete mesh removal with no repair of their hernia, 4 (14%) patients had a hybrid mesh implanted, and 1 (4%) patient had their hernia repaired with a different material of synthetic mesh.

The average postoperative pain score upon initial short-term follow up was 4.4/10 (range 1–10). The average time to short-term follow up was 11 days (range 1 day–21 days). Pain score on long-term follow up was 3.4/10 (range 0–8) with an average follow-up time of 2.3 years (range 1.8 months–6.2 years) (Table 4). Four patients (14%) could not be reached for long-term follow up. No patients experienced bowel obstruction, deep venous thrombosis, pneumonia, peripheral nerve injury, sepsis, pulmonary embolism, urinary tract infection, surgical site infection, ileus, hematoma, or non-healing wound (Table 4).

**TABLE 4:** Post-operative outcomes of patients that underwent complete mesh removal due to mesh implant illness (MII).

	MII (N = 28)
Hospital Length of Stay, mean (range)	2.8 (2–5)
Complications	
Pain requiring intervention	2
Urinary retention	1
Seroma	1
Post-operative pain at short-term followup, average	4.4/10
Postoperative pain at long-term followup, average	3.4/10

After mesh removal, 19/28 (68%) patients had improvement and/or resolution of their systemic MII symptoms within the first month. Figure 2B shows resolution of rashes after mesh removal from an inguinal hernia repair. Upon long-term followup, 18/28 (64%) had resolution of their MII symptoms.

## Discussion

To date, mesh-related complications following inguinal hernia repair have been termed post-inguinal herniorrhaphy chronic pain, often due to mechanical complications, such as meshoma, mesh erosion, and nerve entrapment (17). We present a new subset of patients with mesh-related complications who present with a wide syndrome of non-mechanical systemic reactions to their mesh implant consistent with ASIA or Shoenfeld’s syndrome (7, 8). We term this sub-population of ASIA as patients with mesh implant illness (MII).

It is unclear why a patient may develop MII. Some have categorized these systemic reactions to implants as mediated by a foreign body reaction to the implant, an upregulation in systemic inflammatory markers in response to the implant, a response to the *in vivo* degradation and absorption of the implant, and/or being a high responder to the implant (6, 18). Meanwhile, there is no objective proof that any of these mechanisms are the underlying causes of ASIA (18). *In vitro* trials by Schachtrupp et al., show markedly disparate responses in monocyte reaction to polypropylene mesh (6). While these trials did not extend to the *in vivo* or clinical setting, they propose a monocyte-macrophage response to be contributing to the variable response to implants. Studies on explanted hernia mesh have shown varying degrees of chemical degradation of the implant, suggesting that mesh is not an inert implant in all patients (19). Moreover, we have previously analyzed the clinical significance of explanted mesh pathology evaluation between mesh reaction and non-reaction groups and have found them to be similar (20). In both groups, commonly noted pathology findings included foreign-body reaction, fibrosis, and chronic inflammation (20). At this time, we do not have enough studies to define MII or ASIA to be due to any single or series of abnormalities. We recommend research into more detailed immunologic and inflammatory responses at the tissue level of explanted mesh in patients with suspected MII or ASIA.

In our practice, we see this variance in response to mesh implantation clinically. That is, though most patients have positive outcomes after their hernia repair with mesh, there is a subset of patients who exhibit severe systemic responses after hernia mesh implantation, such as fatigue, bloating, body swelling, joint pain, rash, headaches, fevers, and fibromyalgia (Table 3). In our study, we noted mesh reactions in patients with polypropylene (71%) as well as other materials, such as polyester (7%), cadaveric tissue (11%), and possibly ePTFE (11%) (Table 5). Meanwhile, the *in vitro* study looking at blood monocytes showed reactions primarily to polypropylene mesh (6).

**TABLE 5:** Mesh material removed in patients that underwent mesh removal due to mesh implant illness (MII) or other reasons (non-MII) show no significant difference (*p* < 0.05).

Mesh material removed	MII	Non-MII	*p*
	N = 28	N = 137	
Polypropylene	20 (71%)	107 (78%)	NS
Polypropylene + ePTFE	3 (11%)	12 (9%)	NS
Polyester	2 (7%)	4 (3%)	NS
Hybrid	2 (7%)	3 (2%)	NS
Biologic	1 (4%)	3 (2%)	NS
Polypropylene + Hybrid	0 (0%)	1 (1%)	NS
ePTFE	0 (0%)	6 (4%)	NS
Unknown	0 (0%)	1 (1%)	NS

While individual variability seems to be a determinant in MII, factors such as the size and/or number of implanted meshes, i.e., the load of implant on the body, may play a factor in MII and ASIA. In one study, the severity of oxidative stress and immunologic reaction to polypropylene were directly related to the amount of material implanted per cm^2^ (21). This may explain why 5 (18%) of our patients expressed MII symptoms only after multiple mesh repairs were performed, a larger mesh was placed, and/or after exposure to other implants, such as breast implants and dental implants. This suggests that the amount of foreign body implants, as well as the quality and quantity of the implant, may contribute to an augmented inflammatory and/or immune response in certain patients.

The systemic inflammatory symptoms observed in our patients with MII are consistent with that described in the literature on silicone breast implants (22). Breast implants were introduced to the U.S. market in 1962. In 1980, there was a concern that silicone-based breast implants were responsible for systemic autoimmune disorders, including fibromyalgia, rheumatoid arthritis, lupus, and other connective tissue diseases (23). Due to these concerns, a moratorium on silicone implants was issued in 1992 (21). Further studies at the time failed to confirm a direct association between the silicone breast implants and these systemic symptoms. As a result, the moratorium was lifted in 1999, with the FDA approving two silicone-based implants. As of 2011, the FDA maintains the position that current evidence does not definitively support these systemic complications, lacking power and long-term data (23). More recently, a large population after-market study indeed showed higher risk of serious illness in patients with silicone-based breast implants (22). This has been termed by various groups as silicone implant incompatibility syndrome or, more simply, breast implant illness (BII). BII is now considered a subset of ASIA/Shoenfeld’s syndrome (9–14). As of September 2022, the FDA has issued a safety statement confirming reports of squamous cell carcinoma and various lymphomas in the scar tissues (capsule) that forms around breast implants (24). Some suggest these underlying incidents are related to autoimmunity hyperstimulation by the implants (25, 26).

There is no consensus on the treatment of patients with MII. In our practice, we have taken several different approaches in regards to treating our population of patients with suspected MII. All patients underwent complete mesh removal. It is very important that the suspected implant is fully removed, as partial mesh removal, which may be appropriate for some patients with post-herniorrhaphy chronic pain, is an inadequate procedure for patients with suspected MII. The treatment plan should be carefully determined preoperatively. In our practice, we had 16 (57%) patients undergo a non-mesh tissue-based hernia repair, 7 (25%) required no repair of their hernia, and 4 (14%) patients had their hernia repaired using a hybrid mesh of biologic with a small percentage of permanent suture. We did have one patient who had their hernia repaired with polyester mesh after showing reaction to polypropylene.

In retrospect, we do not recommend replacing one permanent synthetic implant with another in these patients. Based on our experience and also the findings of this study, we recommend erring on preventing implantation of any other forms of synthetic or permanent mesh upon initial mesh removal. However, in some situations, it is not technically possible to complete a mesh removal operation without reinserting some sort of mesh. In those situations where it is absolutely necessary to use an implant, we recommend using an implant with low inflammatory potential, such as a pure biologic mesh or a hybrid mesh with a predominance of biologic tissue. Though unproven, there are theories that such mesh types that have a lower inflammatory potential than standard synthetic and permanent meshes may be less likely to elicit ASIA. That said, 11% of our patients in this study developed MII after implantation of biologic mesh. At this time, we cannot make judgements about the relationship between the type of mesh and risk of MII. Further studies with a larger sample size may be able to shed light on this relationship.

The outcomes from the use of permanent suture, such as polypropylene, polyester, nylon, or PTFE, is unclear in these patients. Though it is considered standard of care for hernia repairs to use permanent suture, it is unclear if the sutures themselves may elicit a reaction. In our study, two patients who had MII underwent mesh removal and tissue-based hernia repair with polyester and polypropylene. Though both improved after mesh removal, they both required removal of their permanent sutures in order to be cured of their ASIA symptoms, showing that in some patients, even the use of permanent sutures may induce an abnormal systemic reaction.

Furthermore, we noticed our mesh reaction population included 3 patients (11%) with a history of an autoimmune disorder and 11 patients (39%) with a history of multiple allergies to either food or medications. Although patients with suspected MII were almost two times more likely to have a history of autoimmune disease, 6% of non-MII patients also had a history of autoimmune disease. Thus, patients with autoimmune diseases can safely have mesh implants without MII. In certain circumstances, we conduct allergy testing and skin patch testing on patients to help determine to what mesh or sutures they may react. That said, at this time, allergy testing is not considered standard of care as we have shown the results in our experience to be inaccurate with low sensitivity (27).

We aim to provide insight based on our experiences into the presentation and treatment options of this subset of patients experiencing MII after mesh-based hernia repair. In patients who we suspect to have MII, we perform complete mesh removal and limit the tendency toward further mesh use. However, our practice and knowledge about this entity is currently evolving. There remains much to be studied about this subset of patients and the cause of their reaction, as we do not know enough about why patients develop ASIA or MII, nor which patients are likely to develop these systemic reactions to their implants in order to help prevent this life-altering problem. Further studies are also needed to develop an algorithm and/or diagnostic tool to determine patients’ susceptibility to MII.

## Data Availability

The raw data supporting the conclusion of this article will be made available by the authors, without undue reservation.
